# Synthesis, characterization, and anti-cancer potential of novel p53-mediated Mdm2 and Pirh2 modulators: an integrated *In silico* and *In vitro* approach

**DOI:** 10.3389/fchem.2024.1366370

**Published:** 2024-07-16

**Authors:** Sarfaraj Niazi, C. P. Kavana, H. K. Aishwarya, Chandan Dharmashekar, Anisha Jain, Tanveer A. Wani, Chandan Shivamallu, Madhusudan N. Purohit, Shiva Prasad Kollur

**Affiliations:** ^1^ Department of Pharmaceutical Chemistry, JSS College of Pharmacy-Mysuru, JSS Academy of Higher Education and Research, Mysuru, India; ^2^ Department of Biotechnology and Bioinformatics, JSS Academy of Higher Education and Research, Mysuru, India; ^3^ Department of Microbiology, JSS Academy of Higher Education and Research, Mysuru, India; ^4^ Department of Pharmaceutical Chemistry, College of Pharmacy, King Saud University, Riyadh, Saudi Arabia; ^5^ School of Physical Sciences, Amrita Vishwa Vidyapeetham, Mysuru Campus, Mysuru, India

**Keywords:** leukemia, MDM2, p53, cancer therapy, drug discovery, small molecule modulators

## Abstract

**Introduction:** Leukemia is a global health concern that requires alternative treatments due to the limitations of the FDA-approved drugs. Our focus is on p53, a crucial tumor suppressor that regulates cell division. It appears possible to stabilize p53 without causing damage to DNA by investigating dual-acting inhibitors that target both ligases. The paper aims to identify small molecule modulators of Mdm2 and Pirh2 by using 3D structural models of p53 residues and to further carry out the synthesis and evaluation of hit candidates for anti-cancer potency by *in vitro* and *in silico* studies.

**Methods:** We synthesized structural analogues of MMs02943764 and MMs03738126 using a 4,5-(substituted) 1,2,4-triazole-3-thiols with 2-chloro N-phenylacetamide in acetone with derivatives of PAA and PCA were followed. Cytotoxicity assays, including MTT, Trypan Blue Exclusion, and MTS assays, were performed on cancer cell lines. Anti-proliferation activity was evaluated using K562 cells. Cell cycle analysis and protein expression studies of p53, Mdm2, and Pirh2 were conducted using flow cytometry.

**Results:** As for results obtained from our previous studies MMs02943764, and MMs03738126 were selected among the best-fit hit molecules whose structural analogues were further subjected to molecular docking and dynamic simulation. Synthesized compounds exhibited potent anti-proliferative effects, with PAC showing significant cytotoxicity against leukemia cells. PAC induced cell cycle arrest and modulated p53, Mdm2, and Pirh2 protein expressions in K562 cells. Molecular docking revealed strong binding affinity of PAC to p53 protein, further confirmed by molecular dynamics simulation.

**Discussion:** The study presents novel anticancer compounds targeting the p53 ubiquitination pathway, exemplified by PAC. Future perspectives involve further optimization and preclinical studies to validate PAC’s potential as an effective anticancer therapy.

## 1 Introduction

Cancer stands as a predominant global cause of mortality, wsitnessing a surge in affected individuals worldwide in recent years. This surge imposes substantial financial, emotional, and physical burdens on individuals, families, communities, and healthcare systems. Among the myriad forms of cancer, leukemia is anticipated to rank as the 11th most prevalent in terms of both incidence and cancer-related mortality. The hematologic malignancy arises from disrupting blood cell production in the bone marrow, where stem cells mature into red and white blood cells and platelets, (Prager et al., 2018). The uncontrolled proliferation of aberrant blood cells impedes the normal developmental process. The etiology of most leukemias remains largely spontaneous, with elusive causative factors. However, scientific investigations indicate a frequent association between these cancers and genetic abnormalities, immunosuppression, as well as exposure to risk factors such as ionizing radiation, carcinogenic chemicals, and oncogenic viruses (Hassan and Seno, 2020). Leukemia is commonly addressed through therapeutic modalities such as chemotherapy, targeted therapy, and stem cell transplantation. Additionally, ongoing research explores the potential of immunotherapy and emerging treatments in leukemia management. Despite the availability of FDA-approved treatments for leukemia, these therapies often result in adverse effects, including fatigue, edema, and muscle cramps. Notable treatments include APR-246 (Eprenetapopt), Nutlins (e.g., RG7112), and Idasanutlin (RG7388).Long-term use of these drugs can lead to hepatic and cardiac problems. Based on their unique mechanisms of action, anticancer drugs have been categorized into four groups: monoclonal antibodies, hormones, anti-tubulin and DNA-interactive hybrids, and antimetabolites. In the ongoing search for less harmful and more effective leukemia treatment strategies, additional therapeutic tumor suppressor paths must be explored (Marei et al., 2021).

Tp53 is known as a tumor suppressor, p53 is useful when it is activated in response to various cellular stressors such as DNA damage or oncogene activity. When it comes to cancer, proper regulation of p53 is essential because it is essential in limiting the unchecked proliferation and survival of cells containing genetic damage or mutations. p53 acts as a protective mechanism, limiting the unregulated proliferation of cells containing genomic defects that may eventually lead to cancer by coordinating cell cycle arrest, DNA repair, and apoptosis (Dittmar and Winklhofer, 2020). The canonical homo-tetrameric p53α protein, widely recognized as the “Guardian of the genome,” emerges as a formidable tumor suppressor encoded by the Tp53 gene. p53 is a transcription factor that regulates numerous cell cycle pathways with previously unheard-of potency in the complex field of leukemia. Its profound ability to regulate essential cellular functions, maintain genomic integrity, and delay the onset of carcinogenesis, makes it indispensable in the context of leukemia. p53 is a major molecular actor that protects cellular homeostasis, and it plays an important function in the treatment of leukemia. There are more than 500 known p53 DNA response elements (REs), which are 20-base pair sequences found in the promoter and enhancer regions of genes (Dittmar and Winklhofer, 2020). These REs are essential for interpreting the complex binding patterns of p53 and coordinating a range of cellular responses that impact outcomes including cell destiny, differentiation, DNA repair, and other physiological processes under stress conditions. The intricate network that controls the Tp53 gene family is influenced by microRNAs (miRNAs), which are crucial for maintaining the integrity of the genome. About 60% of coding genes are regulated by miRNAs, which have an impact on mRNA stability and translation. Research indicates that there is an increasing number of miRNAs that either directly or indirectly affect the Tp53 gene family. This intricate circuitry is mostly responsible for tumor prevention and is genetically modifiable. While treatment alternatives using miRNAs could be effective in fighting cancer, they are plagued by problems including poor cellular uptake, which emphasizes the need for efficient delivery methods and close observation for any side effects. This work focuses on the control of p53, which is regulated by several significant post-translational modifications, including phosphorylation and ubiquitination (Shin, 2023; Li and Zhang, 2022). Phosphorylation activates p53 and modifies its structural makeup, whereas ubiquitination is a more complex process that occurs only at lysine residues and impacts the fate of the substrate protein by forming polyubiquitin chains with distinct effects. It is challenging yet essential to comprehend the intricate interactions between p53 and various ubiquitinating E3 ligases, such as Mdm2 and Pirh2, to create effective anticancer drugs (Daks et al., 2022). Even though little research has been done on specific proteins like p53, Mdm2, and Pirh2, a deeper understanding of the protein-protein interaction network offers crucial insights into putative druggable hot spots, facilitating the development of novel modulators for therapeutic interventions against cancer (Daver et al., 2023). The development of dual-acting inhibitors that target both Mdm2 and Pirh2 E3(Ub)-ligases, with a focus on their promiscuous binding, can help overcome resistance and have a synergistic effect on p53 stabilization (Li et al., 2021). The potential of this method for treating cancer without causing DNA damage. This research aims to exploit a variety of chemical libraries and computational drug discovery techniques to create and identify small compounds that target the interactions between Pirh2 and Mdm2-p53.

The *in silico* results obtained from your previous studies Niazi and Purohit, 2015 were taken into consideration for synthesizing the potential anti-cancer molecules were synthesized using the standard protocol (Haronikova et al., 2021). Therefore, in the present investigation we synthesized structural analogues of MMs02943764 and MMs03738126 using a 4,5-(substituted) 1,2,4-triazole-3-thiols with 2-chloro N-phenylacetamide in acetone with derivatives of PAA and PCA were characterized by employing infrared (IR), 1H nuclear magnetic resonance (NMR) and Mass spectral studies. The aim of this study is to identify small molecule modulators capable of targeting Mdm2 and Pirh2, two critical regulators of the tumor suppressor protein p53, utilizing 3D structural models of specific p53 residues. Further synthesized compounds were subjected to *in vitro* activities to evaluate their cytotoxicity profile. A computational docking method and molecular dynamics (MD) simulations are used to validate and modify promising hit candidate, ensuring their structural stability and potential efficacy of a synthesized compound (Devi et al., 2022).

## 2 Materials and methods

### 2.1 Chemicals

Chemical agents used in the present study included (DMEM and DMSO; S.D. Fine chemicals Ltd., Bengaluru, India), (Trypsin-EDTA Solution; SRL Chemicals, Mumbai, India), (FBS; Sigma, Bengaluru, India), (Camptothecin; Sigma, Bengaluru, India), (Antibodies; Abcam, Waltham, Boston, United States), (37°C incubator with humidified atmosphere of CO_2_; Healforce, China), (Cell line: K562—procured from NCCS, Pune, India). HT microplate spectrophotometer reader was purchased from BioTek (Gujarat, India).

### 2.2 Synthesis of structural analogues of the MMs02943764 and MMs03738126

The structural analogues of MMs02943764 and MMs03738126 consisting of 1, 2, 4 triazole scaffold derived from molecular docking were synthesized as per the Scheme-1 and molecular physicochemical properties of 1, 2, 4-triazole derivatives are presented in (Table 1).

**TABLE 1:** Molecular physicochemical properties calculated for the synthesised 1, 2, 4-triazole analogues of PAA and PCA.

Compound code	miLogP	TPSA (A^2^)	No. of atoms	HBA	HBD	Lipinski’s violations	Rotatable bonds	Volume (^A3^)
PAC, 8(a)	4.89	69.05	31	6	1	1	8	380.96
PAA, 8(e)	4.21	69.05	30	6	1	0	8	367.43

#### 2.2.1 General experimental procedure for synthesizing 2-(4, 5-substituted) 1, 2, 4-triazole-3-ylthio)-N-(p-substituted)-phenyl acetamide derivatives of PAA and PCA

To a solution of appropriate 4, 5-(substituted) 1, 2, 4-triazole-3-thiols, (0.001 mol) in 30 mL acetone, appropriate 2-chloro N-phenylacetamide, (0.001 mol) was added. The mixture was vortexed under reflux condition for 3–5 h in the presence of anhydrous K_2_CO_3_ (0.005 mol, 0.69 g). The reaction was monitored by TLC using a mixture of ethyl acetate and hexane in 1:1 ratio as the mobile phase. The reaction mixture was filtered and poured into a beaker containing ice cubes. The precipitate formed was filtered, rinsed with cold water and recrystallized from ethanol to obtain 2-(4, 5substituted)-1, 2, 4-triazole-3-ylthio)-N-(p-substituted)-phenyl acetamide derivatives (Ali et al., 2021).

### 2.3 Characterization of synthesized compounds

Perkin Elmer FT-IR type 1650 spectrophotometer was used to record the infrared spectra of the synthesized compounds within the range of 4000–400 cm^−1^ considering the Potassium bromide pellets. The 1H-NMR was analyzed using a Bruker AV-500 spectrometer and DMSO-d_6_ was used as a solvent and tetramethylsilane was used as internal standard. The mass spectroscopy of synthesized compounds was analyzed using Agilent technologies (HP) 5973 mass spectrometer with an ionization potential of 70 eV.

### 2.4 Cytotoxicity assay

#### 2.4.1 MTT assay

The evaluation of cytotoxicity was performed using MTT assay. The K562 cells were plated in a 96-well culture plate with various concentrations (25 µM–160 µM) of the methanol extract and fractions. The cultured plates were incubated for 24, 48, and 72 h at 37°C and 5% CO_2_. Following incubation, 20 µL MTT solution in phosphate-buffered saline (PBS) was added to each well at a final concentration of 0.5 mg/mL followed by further incubation for 3 h at 37°C. The medium was then removed, and 100 µL DMSO was added to each well for solubilizing the formazan. The absorbance was measured at 490 nm (630 nm as a reference) using an ELISA reader (SkanIt™ Software, Microplate Readers, Thermo Fisher Scientific). Three independent experiments were carried out, and 8 replicates were taken for each experiment. The concentration of the methanol extract and fractions which resulted in a 50% reduction of cell viability, the half maximal inhibitory concentration (IC50 value), was calculated using the following formula: % inhibition = (control abs - sample abs)/(control abs) × 100. Paclitaxel was used as a positive control at the concentration of 0.2–50 μg/mL (Ghasemi et al., 2021).

#### 2.4.2 Trypan blue exclusion assay

To evaluate the antiproliferative effects of the novel PAC and PAA compound, the trypan blue exclusion assay was performed. Cell K562 were seeded into 12-well plates at a density of 2 × 10^4 cells/well. After 24 h, cells were exposed to the tested compounds at concentrations corresponding to their respective IC50 values and then incubated for 72 h. Subsequently, cells were harvested and centrifuged at 500 *g* for 5 min. Following centrifugation, the supernatant was discarded, and the cell sediment was dissolved in 0.2 mL of PBS. Next, 10 µL of the cell solution was mixed with 10 µL of trypan blue dye. Cell counting was performed using an automatic cell counter (Countessa). The data obtained were expressed as the mean ± standard deviation (SD), and the mean percentage of viable cells was calculated from three independent experiments, each performed in triplicate (Tavares-Carreón et al., 2020).

#### 2.4.3 MTS assay

The cytotoxicity of synthesized compounds was evaluated by MTS assay using the K562 cancer cell line. The cancer cells were cultured in DMEM (Gibco, United States) supplemented with 10% FBS (Gibco, United States) at 37°C with 5% CO_2_. Briefly, a total of 2000 K562 cells for each well were cultured overnight in a 96-well plate. Then, the novel PAC and PAA compound or Doxorubicin were added to each well at varied concentrations (0.01 μM, 0.1 μM, 1 μM, 10 μM, 100 μM). After 4 days, MTS and PMS were added to the cell culture and incubated at 37°C for 2–3 h. The absorbance was measured at 490 nm to quantify the viable cells. The growth ratio of treated cells was calculated by comparing the absorbance to the non-treated cells (Ma et al., 2022).

### 2.5 Anti-proliferation activity

The K562 cells (1 × 10^6^ cells/well) were procured from ATCC. The stock cells were cultured in DMEM which is supplemented with inactivated FBS (10%), penicillin (1%; 100 IU/mL), and streptomycin (100 μg/mL) was added in a humidified atmosphere containing CO_2_ (5%) at 37°C until confluent. The cells are dissociated with TPVG solution having a composition of trypsin (0.2%), EDTA (0.02%), and glucose (0.05%) dissolved in PBS. The viability of the cells is then checked using trypan blue and centrifuged. Further, 5.0 × 10^4^ cells/well were seeded in a 96 well plate and finally incubated for 24 h in CO_2_ (5%) incubator at 37°C.

The monolayered cell culture was trypsinised. The cell count was brought to 5.0 × 10^5^ cells/mL by using the medium containing 10% FBS. Next, 100 µL of diluted cell suspension was added to each well of the microtiter plates. After 24 h, when the monolayer is formed, the supernatant is removed. 100 μL of different experimental compounds added to the wells containing the monolayer. These plates were kept for incubation in CO_2_ (5%) incubator for 24 h at 37°C and cells will be periodically checked for physical changes such as granularity, shrinkage, and swelling. After 24 h of incubation, the sample solution was removed from the wells. 100 μL of MTT (5 mg/10 mL of MTT in PBS) was added to the wells. The plates were gently shaken and incubated for 4 h at 37°C under the CO_2_ (5%) environment. The supernatant was removed and DMSO (100 µL) was added. The plates were again gently shaken to solubilize the formazan produced in the viable cells. The absorbance values were taken from the microplate reader at a wavelength of 590 nm. The percentage growth inhibition has been calculated as per the protocol (Tian et al., 2022).

### 2.6 Cell cycle analysis by flow cytometry

To analyze the cell cycle phase distribution, K562 cells (1 × 106 cells/well) were seeded in a 6- well plate for 48 h and then exposed to the experimental compounds (PAC (68.44 μM) and standard drug, Calprotectin (25 μM)) and control. After incubation for 48 h, the untreated and treated cells were rinsed 2x with PBS. The cells are then fixed in 70% ethanol at −20°C for 30 min. The fixed cells were again washed with PBS and 50 μL of RNase solution was added. 400 μL of PI solution/million cells added directly to the cell’s RNase A suspension. Mixed well and incubated for 20–30 min at room temperature in the absence of light. The cell cycle was measured with BD FACSCalibur™ and the percentages of cells (10,000 cells in total) in the different phases (G1, S, and G2) were calculated by the Cell Quest software (BD Biosciences) (Bunney et al., 2017).

### 2.7 p53, Mdm2, and Pirh2 protein expression studies using flow cytometry

The K562 cells (1 × 10^6^ cells/well) was seeded in a 6-well plate and incubated under CO_2_ atmosphere at 37°C for 24 h. After 24 h, the cells were cultured in a medium containing required concentrations of experimental compounds and controls for 24 h. Cells were then washed twice with PBS and 200 μL of trypsin-EDTA solution was added. The mixture was then incubated at 37°C for 3–4 min. To this, 2 mL culture medium was added, and the cells were harvested directly into the 12 × 75 mm polystyrene tubes followed by centrifugation and 1xPBS wash. PBS was decanted completely, and the cells were fixed with 70% chilled ethanol followed by incubation at −20°C for 30 min 20 μL of primary antibody (p53/Mdm2/Pirh2) was added (Parrales et al., 2016). The cells were washed with 2xPBS and treated with 20 µL of secondary fluorescent antibody Phycoerythrin (PE) and incubated for 30 min at room temperature in dark condition. The cells were then rinsed with 1xPBS to remove unbound secondary antibody and re-suspended in the 0.5 mL of PBS. The cells were analyzed for p53, Mdm2, and Pirh2 proteins expression by using BD FACSCalibur™. At least ten thousand cells were counted for each sample (Bunney et al., 2017).

### 2.8 Computational analysis

#### 2.8.1 Ligand preparation

The 3D structures of the predicted bioactive ligand (PAC) were meticulously prepared utilizing the Ligand Prep module within the Schrödinger suite. Through this process, the ligands were subjected to minimization procedures to optimize their conformations. The resulting refined ligands were subsequently assessed for their binding affinity through molecular docking analyses (Bunney et al., 2017).

#### 2.8.2 Protein preparation and binding site analysis

The Structure of Human MDM2 Protein (PDB ID: 3LBK) was retrieved from the Protein Data Bank and prepared using Maestro’s Protein Preparation Wizard. This involved meticulous refinement, including the removal of atomic clashes, water molecules, and unnecessary atoms, as well as the addition of missing atoms and hydrogen. The Sitemap tool in Maestro was then employed to generate the binding site, facilitating precise analysis of potential targets within the TP53 pathway. These procedures, employing advanced computational tools, establish a robust foundation for elucidating protein interactions and exploring therapeutic strategies within the TP53 pathway (Hatami et al., 2023).

#### 2.8.3 Molecular docking

The docking study utilized the Glide (Grid-based Ligand Docking with Energetics) protocol, as described by Friesner et al., in 2004. A two-tier docking approach was employed, involving standard precision (SP) and extra precision (XP). The compounds were docked using Glide, and the resulting conformers were systematically evaluated using the Glide score reprise (Ban et al., 2018).

#### 2.8.4 Molecular dynamic simulation

The compounds underwent filtration based on criteria such as Glide score (Kcal/mol), protein-ligand non-bonded interactions, ligand-active site complementarity, and a review of relevant literature. The top complex, selected through this screening process, underwent further scrutiny via MD Simulations utilizing the Desmond module within the Schrödinger Suite 2022-23 (https://newsite.schrodinger.com/platform/products/maestro/). The analysis aimed to evaluate intermolecular interactions and the stability of the complex across varying time scales. For system construction, the TIP3P solvent model and an orthorhombic water box shape were chosen, with the addition of counter ions for system neutralization. The resulting model system was loaded into the molecular dynamics work panel, and the simulation run time was set at 200 ns. Post-simulation, the complexes were analyzed using the simulation interaction diagram panel within the Desmond module (Du et al., 2016; Kaushik et al., 2018).

### 2.9 Statistical analysis

All experiments were conducted in triplicate, data were presented as mean ± standard error of the mean (SEM). Student’s t-test was employed for comparing between two groups in the *in vitro* assay. To assess the statistical significance of the data, one-way analysis of variance (ANOVA) was utilized, with a significance level set at *p* < 0.05. Statistical analysis was performed using GraphPad Prism 8.0 (Aly et al., 2020).

## 3 Results and discussion

### 3.1 Synthesis and characterization of compounds consisting of 1, 2, 4triazole scaffolds of PAA and PAC

The free mercapto group present in the 1,2,4-triazole intermediate readily reacts with appropriate chloroacetamide under dry conditions to yield the final compounds of PAA and PAC. The synthesized compounds of PAA and PAC were characterized by determining physical constants (Rf values and melting points) and different spectroscopic methods such as IR, ^1^H NMR, 13C NMR, Mass spectroscopy and single crystal XRD. The experimental physical data of the synthesized compounds of PAA and PAC is provided in Table 2 and the calculated values of molecular physicochemical properties are shown in Table 1 (see materials and methods section).

**TABLE 2:** Experimental physical data of the synthesized 1, 2, 4 triazole.

Compound code	Molecular formula (^S^)	M.W (gm)	M.P. (°C)	R_*f*_	% yield
PAC, 8(a)	C_25_H_24_N_4_O_3_	450.94	164	0.37	41.73
PAA, 8(e)	C_23_H_20_N_4_O_2_	416.00	111	0.34	58.32

IR spectra of all the synthesized compounds of PAA and PAC showed stretching absorption bands around regions 1550–1570 cm^−1^ due to N=N and 1560–1640 cm^−1^ due to C=N functions confirming the presence of 1,2,4-triazole ring. The absence of absorption band around 2576 cm^−1^ which corresponds to–SH group and retaining of absorption band −750 cm^−1^ corresponded to C-S function confirms the coupling of triazole ring with acetamides via. reactive -SH group. The spectral characterization by ^1^H NMR spectra of all the synthesized compounds of PAA and PAC showed the chemical shift signals in the range of δ 6.77–6.9 ppm (Ar-H) resonated as multiplet for aromatic protons. The NH proton corresponded to the amide group resonated as a singlet and showed the chemical shift signal around 10.3 ppm. Also, the chemical shift signals at δ 4.1 ppm and δ 5.0 ppm resonated singlet peaks corresponded to that of α-proteins S-CH_2_- C=O and O-CH_2_-C=N respectively. Mass spectrum of the compounds of PAA and PAC showed intense molecular ion peak [M]^+^ and [M+1]^+^ peak for chloro-substituted compounds which are consistent to with respective molecular weights. All the screenshots of the spectral data collected for the compounds of PAA and PAC are provided as Figures 2–7. Analysis of overall spectroscopic data for the compounds of PAA and PAC suggests the formation of desired chemical scaffolds (Table 2) (Volkov et al., 2022).

**FIGURE 1:**
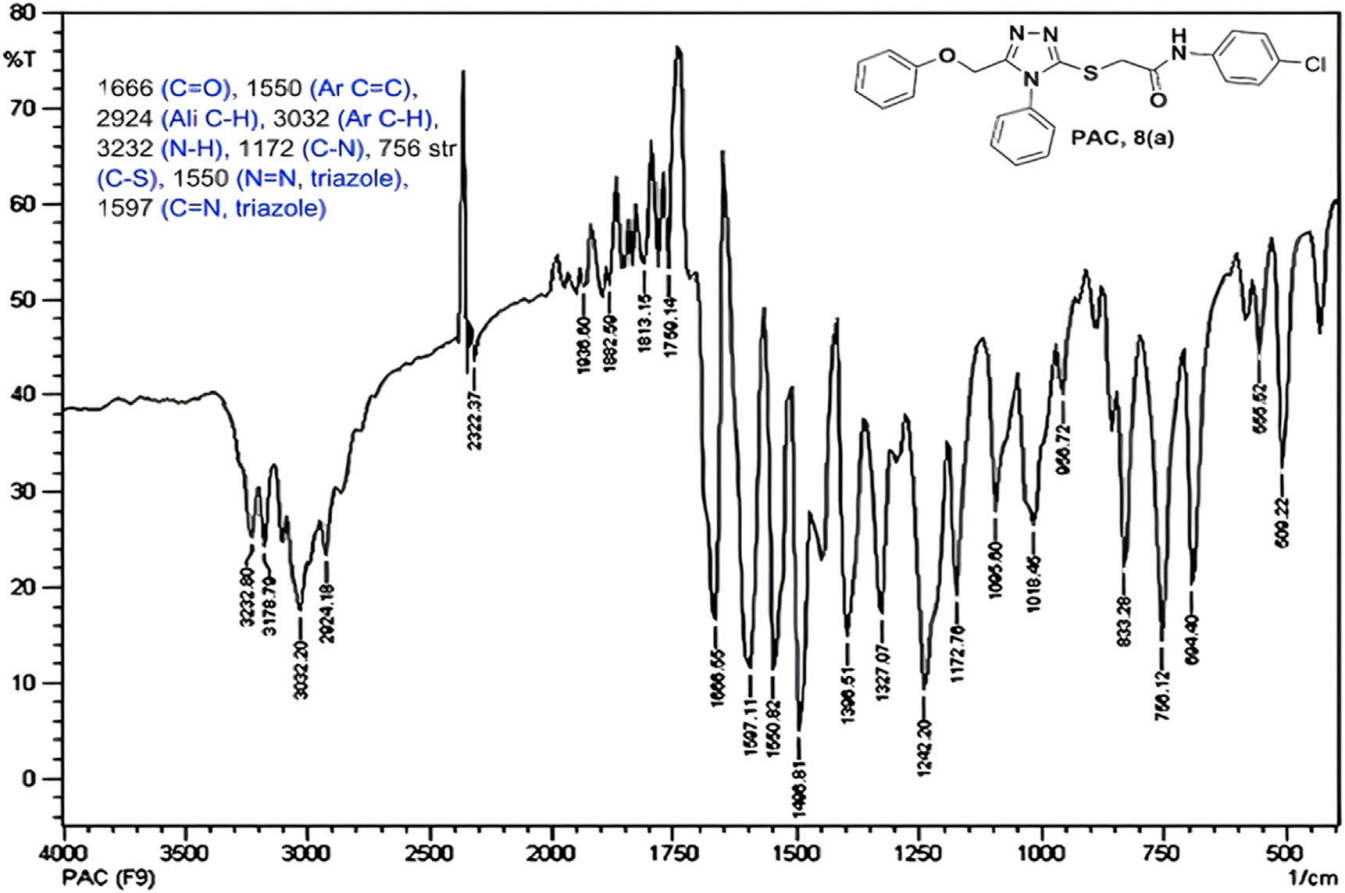
IR spectrum of PAC.

**FIGURE 2:**
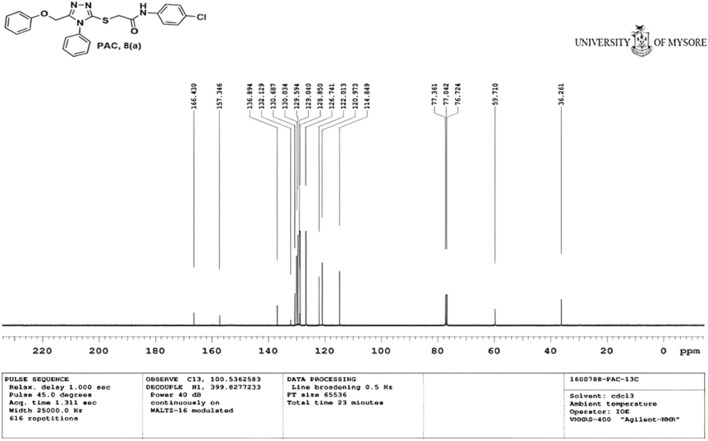
^1^H NMR spectrum of PAC.

**FIGURE 3:**
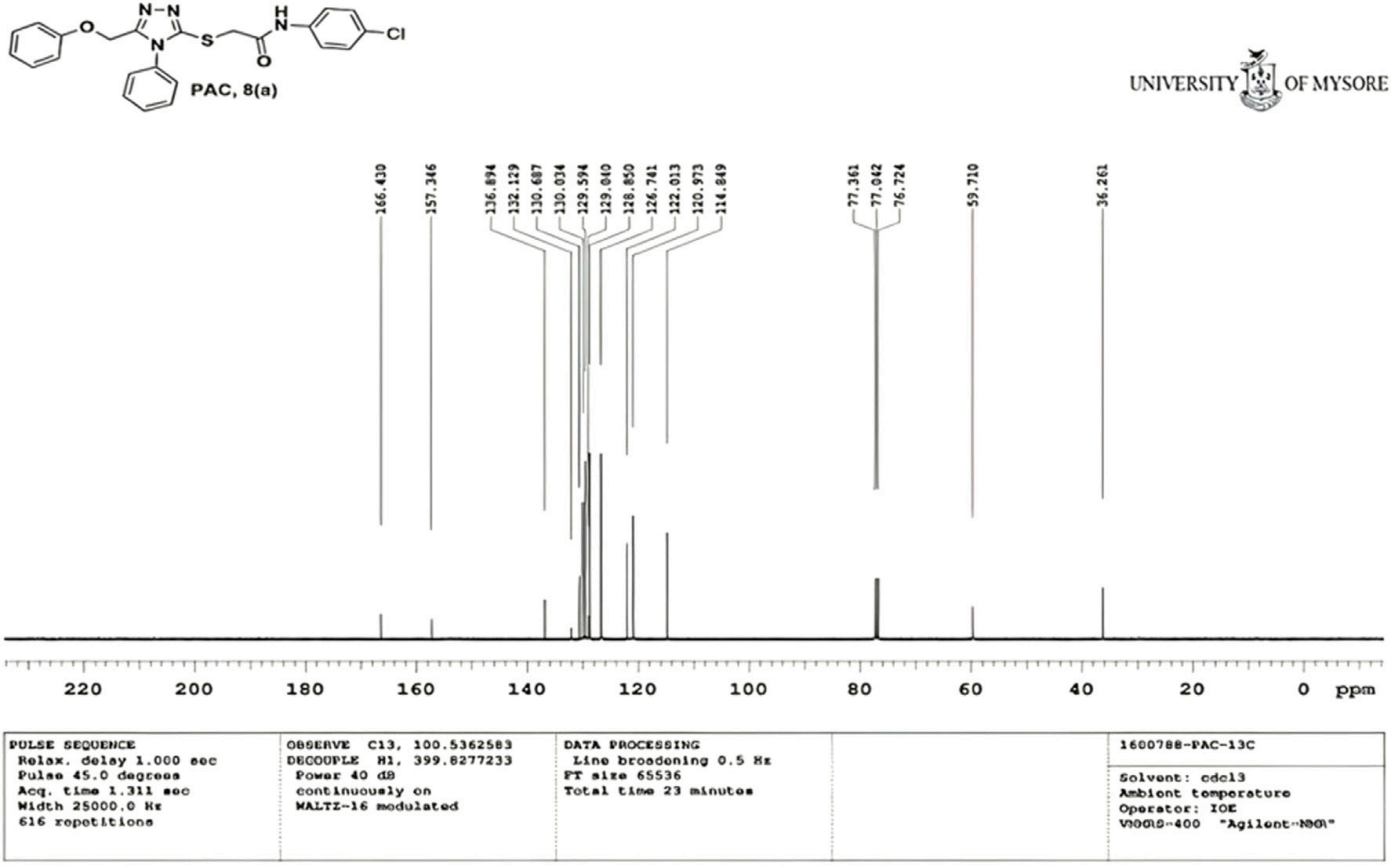
^13^C NMR spectrum of PAC.

**FIGURE 4:**
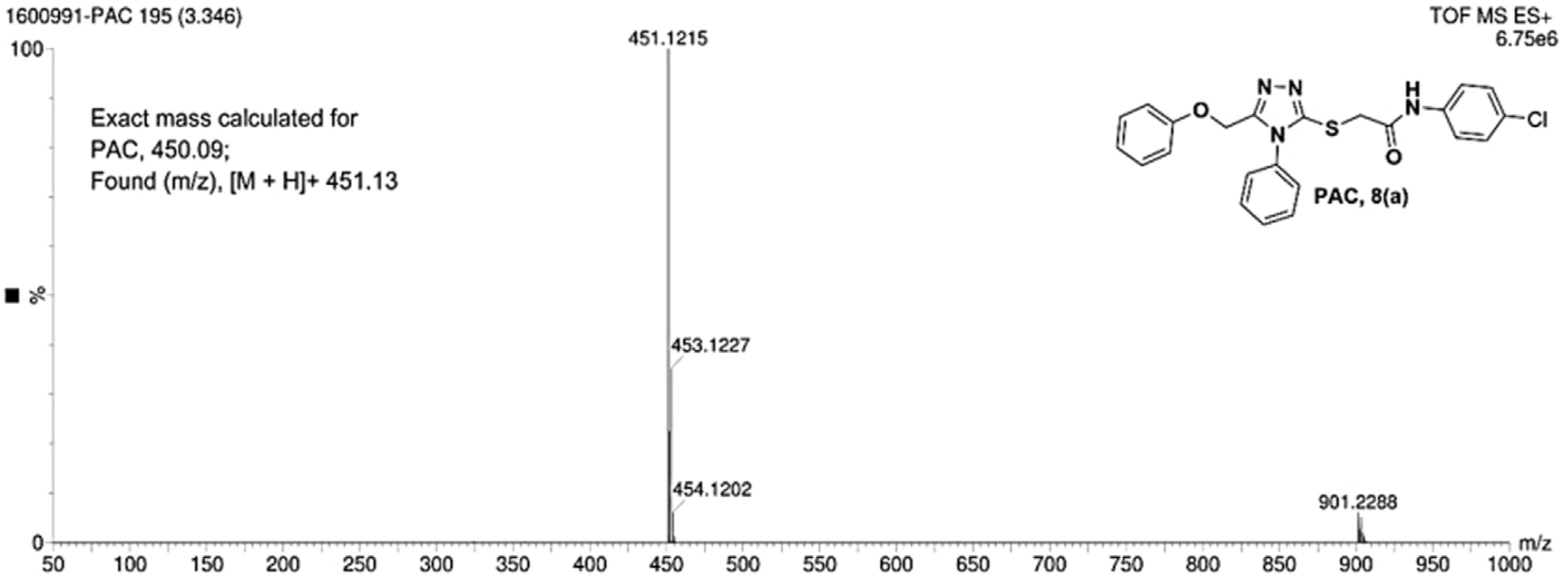
Mass spectrum of PAC.

N-(4-chloro phenyl)-2-((5-(phenoxy methyl)-4-phenyl-4H-1, 2, 4-triazol-3 yl)thio) acetamide (8a; PAC): IR (νmax, cm^-1^): 1666 (C=O), 2924 (Ali C-H), 3032 (Ar C-H), 3232 (N-H), 1172 (C-N), 756 str (C-Cl), 1550 (N=N, triazole), 1597 (C=N, triazole); ^1^H NMR (400 MHz, CDCl3) δ 10.482 (s, -NH-, ^1^H), 7.561-6.844 (m, Ar-CH-, 14H), 5.053 (s, O-CH2-, 2H) 3.962 (s, S-CH2-, 2H); 13C NMR (100 MHz, CDCl3) δ 166.43, 157.34, 136.89, 132.12, 130.68, 130.03, 129.59, 129.07, 128.85, 126.74, 122.01,120.97, 114.84, 59.71, 36.26; Mass: Exact mass calculated for C_23_H_19_C_l_N_4_O_2_S, 450.09; found (m/z), 451.13 [M + H] ^+^ (Figures 1–4).

**FIGURE 5:**
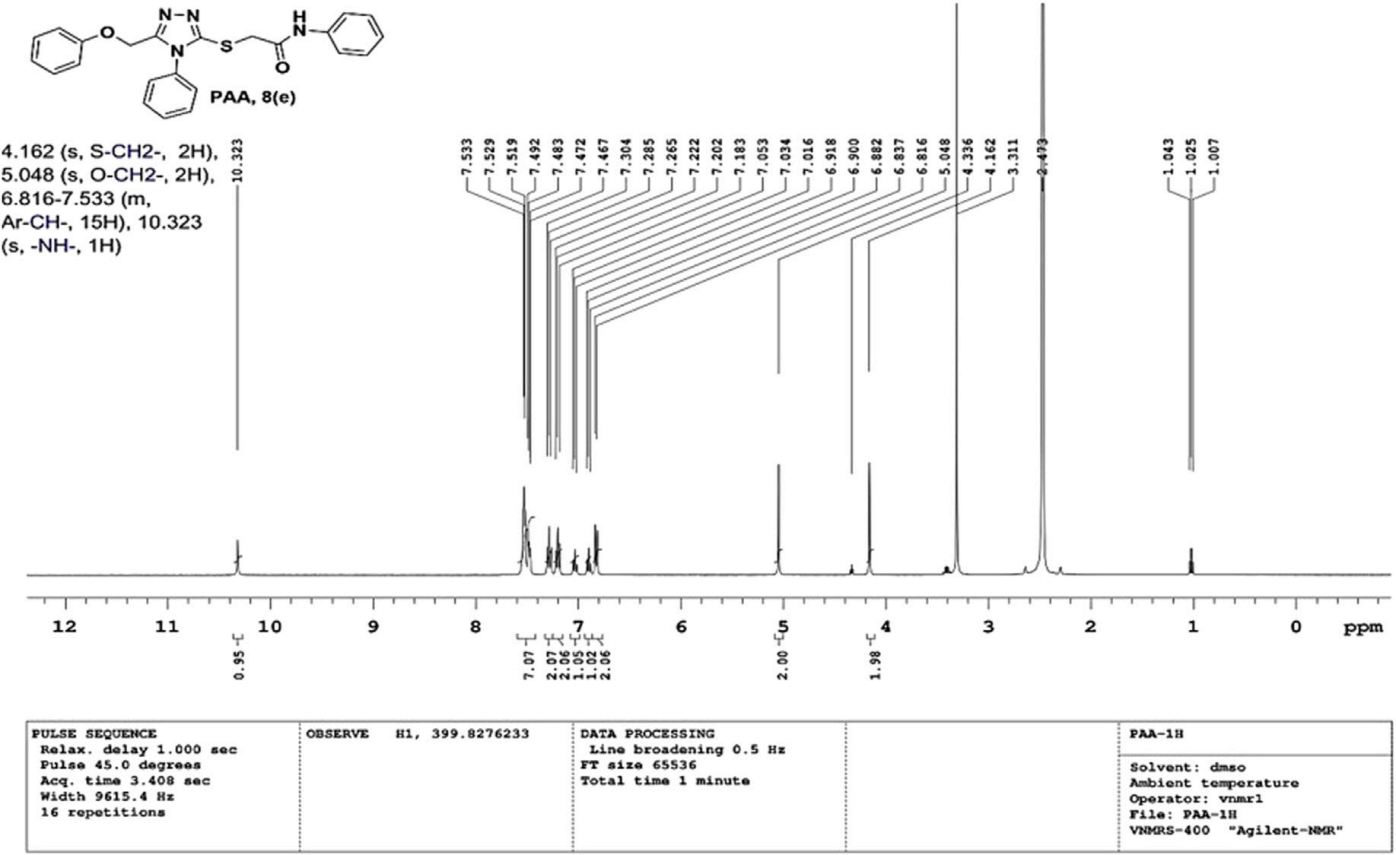
^1^H NMR spectrum of PAA.

**FIGURE 6:**
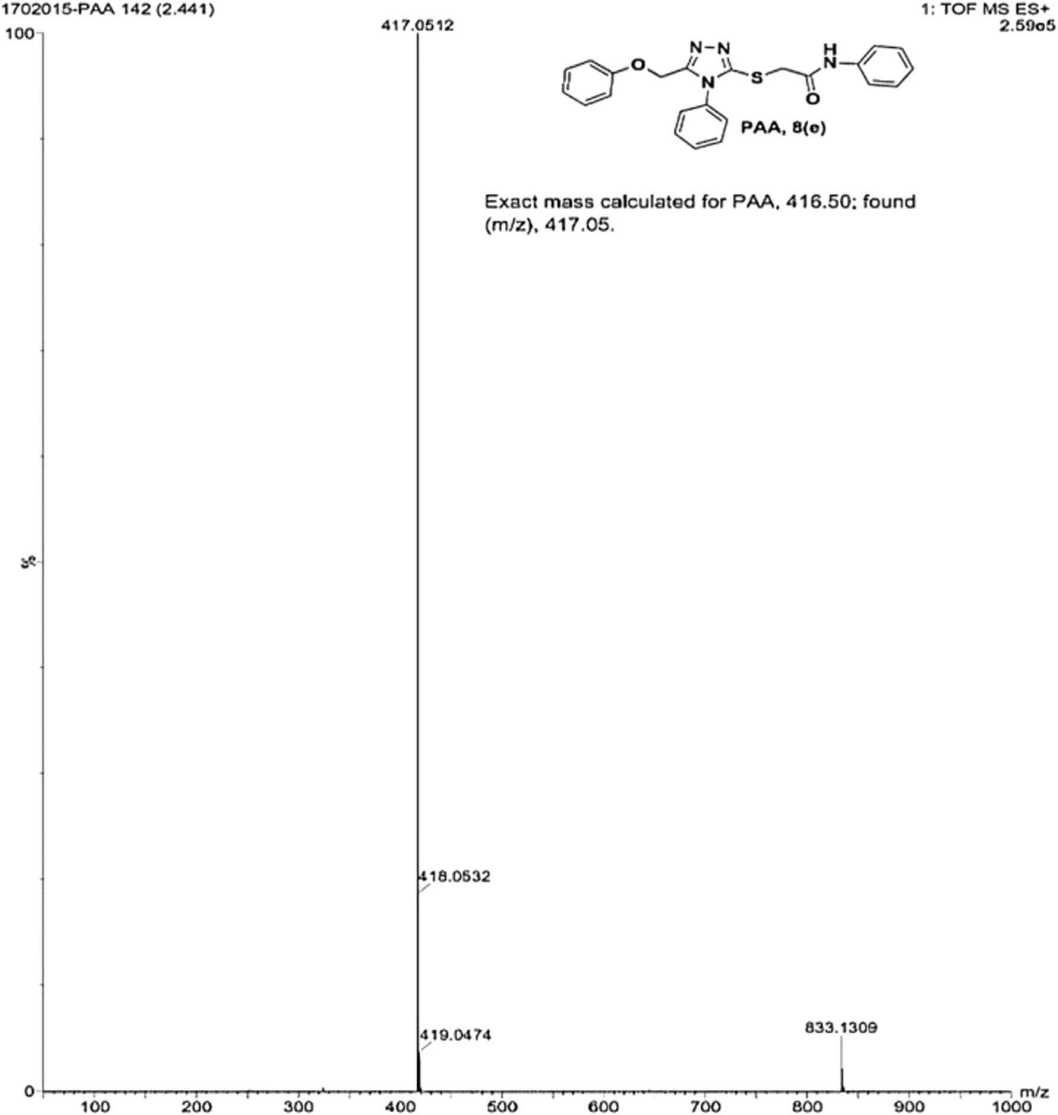
Mass spectrum of PAA.

2-((5-(phenoxy methyl)-4-phenyl-4H-1, 2, 4-triazol-3-yl)thio)-N-phenyl acetamide (8e; PAA): IR (νmax, cm^−1^): 1666 (C=O), 1550 (Ar C=C), 2931 (Ali C-H), 3055 (Ar C-H), 3240 (N-H), 1172 (C-N),763 str (C-S), 1550 (N=N, triazole), 1597 (C=N, triazole); ^1^H-NMR (400 MHz, DMSO-d6) δ 10.323 (s,-NH-, ^1^H), 7.533–6.816 (m, Ar-CH-, 15H), 5.048 (s, O-CH2-, 2H), 4.162 (s, S-CH2-, 2H); Mass: Exact mass calculated for C_23_H_20_N_4_O_2_S, 416.50; found (m/z), 417.05 [M+] (Figures 5, 6).

### 3.2 *In vitro* evaluation

#### 3.2.1 MTT assay

**FIGURE 7:**
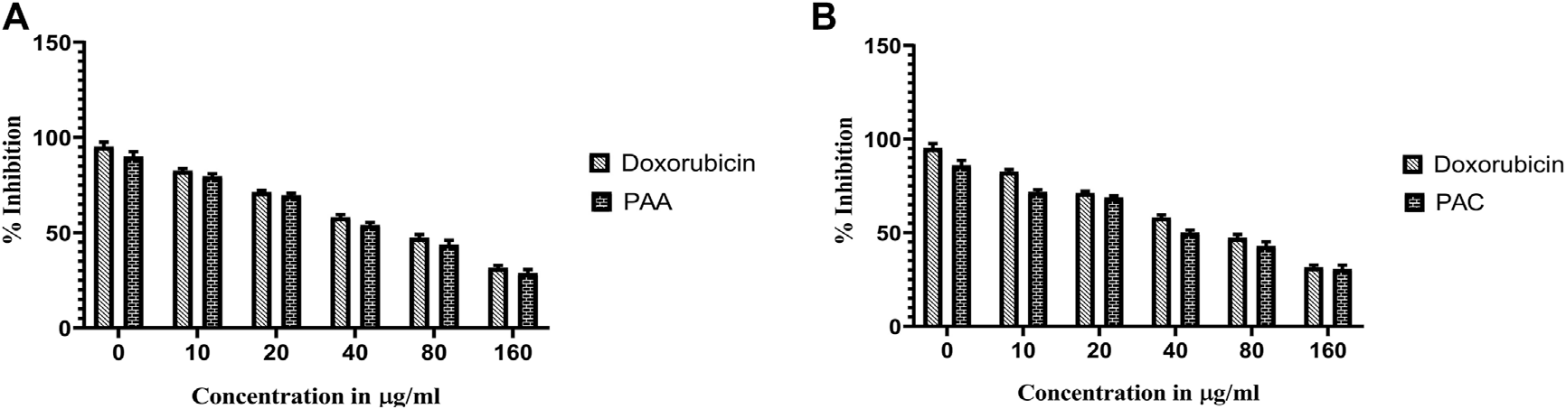
The cell viability inhibition of K562 cell lines against PAC and PAA synthesized compounds for about 48 h using MTT assay. The cell viability inhibition of K562 cell lines upon treatment of **(A)** PAC and **(B)** PAA in concentration dependent manner for about 48 h using MTT assay. One-way ANOVA followed by Dunnett’s multiple comparison test was used to identify significant differences by multiple comparisons. Data are expressed as mean ± SEM (n = 3), ****p < 0.0001 represents the comparison between respective groups with doxorubicin.

The cytotoxicity active of the synthesized PAC and PAA compounds was tested against K562 cell lines using MTT assay. As shown is Figure 7 the PAC shows the better cytotoxic effect when compared to PAA and standard drug doxorubicin in a concentration and time—dependent manner with 48 h of exposure. The results confirm that, upon increase in concentration up to 160 μg/mL could decrease the cell viability (<0.05).

#### 3.2.2 Trypan blue exclusion assay

**FIGURE 8:**
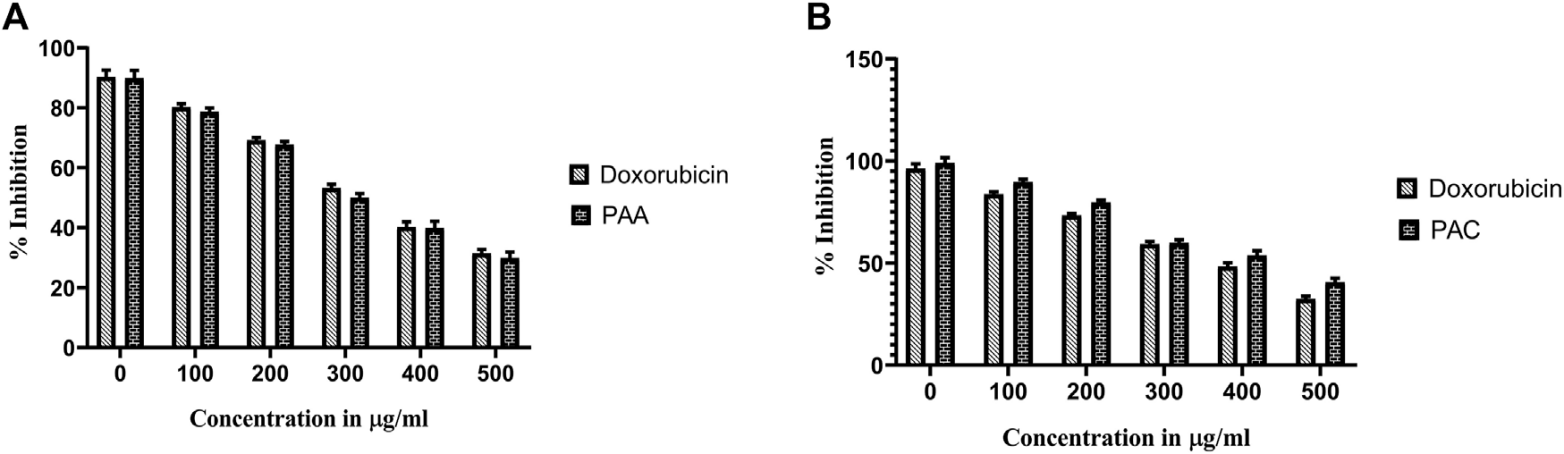
The effect of synthesised PAC **(A)** and PAA **(B)** compounds upon K562 cell lines on total cell number and viability (%) was measured by trypan blue assay. One-way ANOVA followed by Dunnett’s multiple comparison test was used to identify significant differences by multiple comparisons. Data are expressed as mean ± SEM (n = 3), ****p < 0.0001 represents the comparison between respective groups with doxorubicin.

Trypan Blue Exclusion Assay was used to analyse the total viability of cells after exposure of PAC and PAA compound upon IC50 concentration of MTT assay (Table 3). K562 cells were incubated for about 72 h. The PAA compound and standard drug doxorubicin done not show any significantly affect, whereas PAC compounds shows better cell inhibition as shown in Figure 8.

**TABLE 3:** Results of antiproliferation activity of selected compounds performed on the MCF 7, Reh, Nalm6, K562 cell lines.

Compound code	K562 IC50(µM48 h)
Control	-
PAC	35.264
PAA	54.40

#### 3.2.3 MTS assay

**FIGURE 9:**
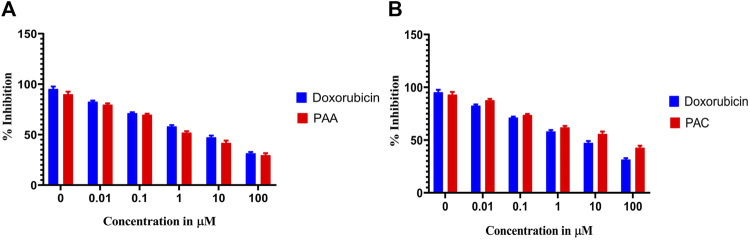
The cell viability inhibition of K562 cell lines against PAC and PAA synthesized compounds for about 48 h using MTS assay. The cell viability inhibition of K562 cell lines upon treatment of **(A)** PAC and **(B)** PAA in concentration dependent manner for about 48 h using MTT assay. One-way ANOVA followed by Dunnett’s multiple comparison test was used to identify significant differences by multiple comparisons. Data are expressed as mean ± SEM (n = 3), ****p < 0.0001 represents the comparison between respective groups with doxorubicin.

**FIGURE 10:**
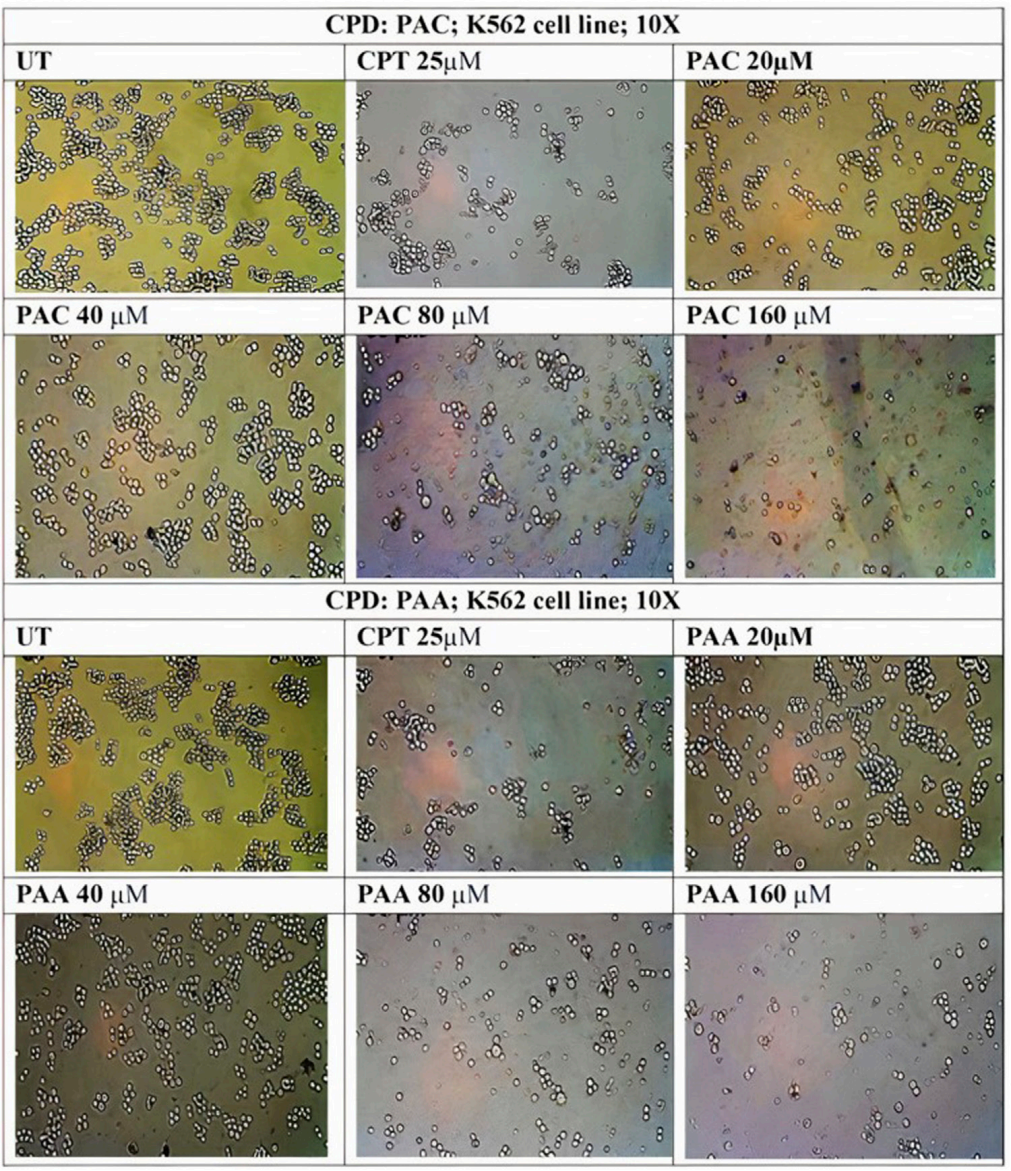
Photomicrographic images of untreated K562 colonies and the drug treatment induced inhibition of proliferation and subsequent inhibition of growth in K562 cells as obtained from MTS assay. Both **(A)** PAC and **(B)** PAA induced the dose dependent inhibition of proliferation in K562 human leukaemia cell lines.

Cytotoxicity active of the synthesized compounds was evaluated by MTS assay using the K562 cancer cell line. The PAC treatment has caused a dramatic fall in the expression levels of both Mdm2 and Pirh2 oncoproteins as compared to the untreated K562 cells. As shown in Figures 9, 10 the PAC shows the better cytotoxic effect when compared to PAA and standard drug doxorubicin in a concentration and time—dependent manner with 48 h of exposure. The results confirm that, upon increase in concentration up to 100 μg/mL could decrease the cell viability (<0.05) (Jiao et al., 2020).

### 3.3 Cell cycle study: PAC induces K562 cell cycle arrest predominantly at the SubG0/G1 and S phases

Since PAC exhibited relatively better anti-proliferation activity in all the tested cancer cell lines, therefore, it was further selected for cell cycle studies. The cell cycle study was conducted on K562 cells in order to decipher the growth phase which is prone to the PAC treatment. The results indicated that the higher percentage of K562 cells are arrested at SubG0/G1 and S phases as evident. A marked increase in the percentage of gated cell populations which are in SubG0/G1, S and G2 phases was detected as compared to that in control K562 p53 null cells. This implies that PAC induces cell cycle arrest predominantly at the SubG0/G1, S and G2 phases. This also suggests an increased level of p53 due to inhibition of Mdm2/Pirh2 by PAC in K562 p53 null cells has occurred thus blocking cell cycle progression since p53 also acts at the G1 checkpoint during G1/S transition phase of the cell cycle and induces the expression of cyclin-dependent kinase (cdk) inhibitor. The cdk inhibitor inactivates the Cdk-G1/S cyclin complex, which later blocks cell cycle progression. These facts suggest that PAC rescues p53 by inhibiting ubiquitination by Mdm2/Pirh2 in K562 p53 null cells (Sane and Rezvani, 2017).

Whereas, in case of cells treated with Camptothecin (CPT; 25 µM), the highest percentage of cells got arrested during G2/M phase (30%) which suggests CPT induces arrest G2/M phase of the cell cycle by a distinct mechanism.

### 3.4 Flow cytometric analysis revealed PAC induces expression of p53 and suppress Mdm2 and Pirh2 proteins expressions

Since PAC and its analogues have been designed *in silico* to modulate the ubiquitination of p53 by both Mdm2 and Pirh2 E3 enzymes, hence its impact on the expression levels of p53, Mdm2 and Pirh2 proteins have been studied by flow cytometry. The expression levels of p53 and Mdm2 and Pirh2 proteins were measured in K562 cells under control and treated conditions. In the histogram graphs Figures 5C–H, the M1 region represents lower expression levels of p53, Mdm2, and Pirh2 protein while the M2 region represents normal to high expression levels.

Generally, K562 cells express a negligible amount of p53, and the results obtained from the flow cytometric analysis has also indicated the lower expression of the p53 tumour suppressor protein under control conditions. Interestingly, exposure of the K562 cells to the indicated concentrations of CPT (reference) and PAC has caused a dramatic increase in the p53 levels. Quantitatively, the exposure of K562 cells to CPT, which is a topoisomerase inhibitor derived from natural source, has triggered ∼10 folds rise in the p53 level, while the PAC treatment has caused ∼4-fold rise in the p53 levels (Figure 7).

**FIGURE 11:**
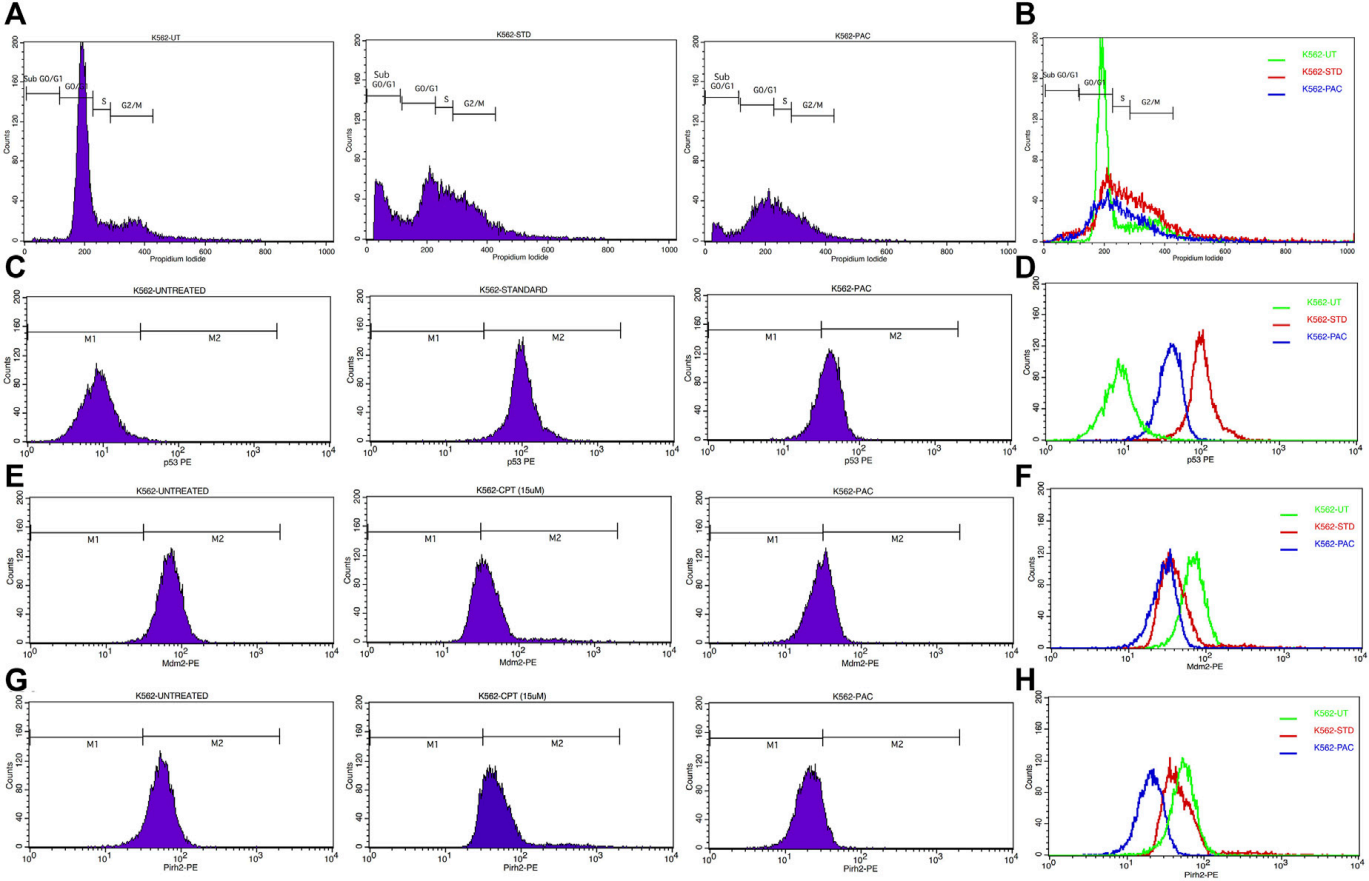
**(A)** CPT and PAC induced K562 cell cycle arrest. Both the synthesized compounds, CPT and PAC were treated against K562 cell lines with different concentrations for about 48 h. The degraded cells were recounted with PI stain during sub G0/G1, S, and G2/M phase using flow cytometry.**(B)** Overlay graphs showing the DNA content of cell cycle progression in K562 leukaemia cells of control (untreated), CPT, and PAC treated groups. The percentage content of DNA in various phases of the cell cycle (G1, S, G2/M and subG1 phase) after 48 h of incubation have been analysed by using Cell Quest software (BD Biosciences). Left panels **(C,E, G)** histograms and the right panels **(D,F, H)** overlay graphs of flow cytometric analysis of p53, Mdm2 and Pirh2 protein expression in K562 cells.

**FIGURE 12:**
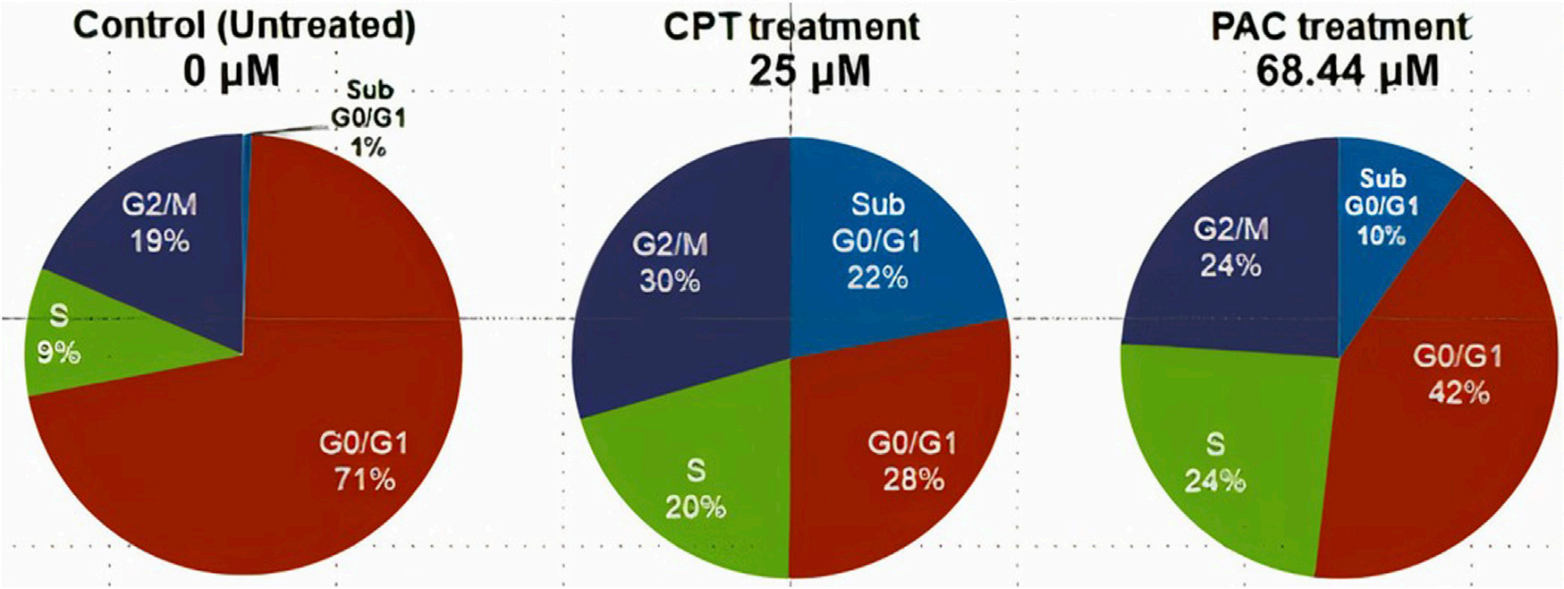
Pie charts illustrating the percentage of gated cells at SubG0/G1, G0/G1, S and G2/M.

**FIGURE 13:**
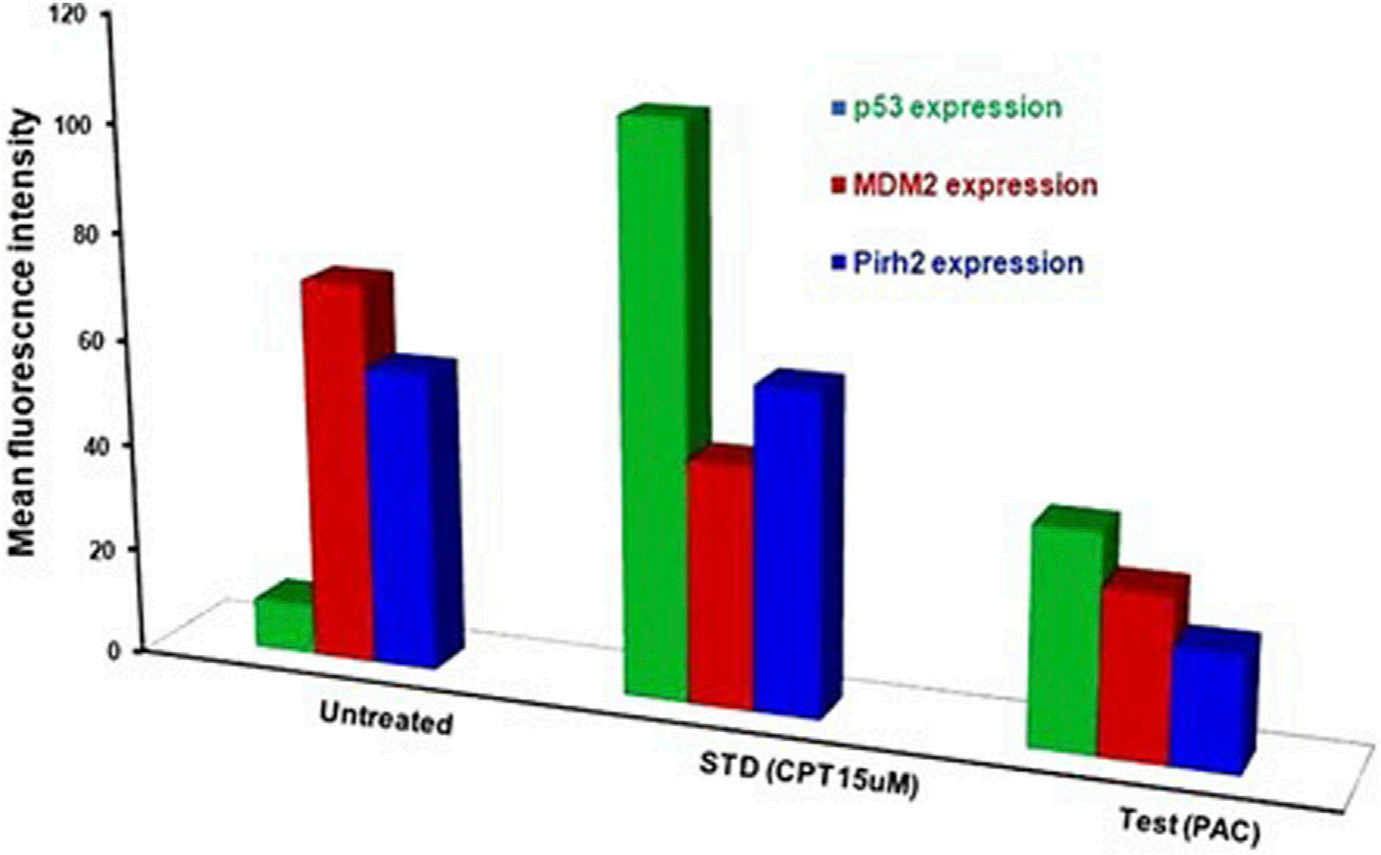
Histograms representing the flow cytometric analysis of p53, Mdm2 and Pirh2 expression levels in control, K562 cells treated with an IC50 concentration of test compound (PAC) and CPT (15 µM). phase of K562 cell proliferation under treated (CPT and PAC) and control conditions.

Generally, the expression level of Mdm2 is not affected in K562 cancer cells (Table 4), and the status Pirh2 expression in K562 cancer cell lines has not been documented till date. However, Mdm2 serves as a primary negative regulator of p53 expression whereas Pirh2 serves as secondary. Hence, it may be presumed that the expression level of Mdm2 is slightly high as compared to the Pirh2. In an agreement, the flow cytometric analysis indicated that Mdm2 expression was slightly high when compared to Pirh2 in control group of K562 cell population (see Figures 11E–H, 13), which reinforces the notion that Mdm2 is a primary negative regulator of p53. On the other hand, CPT, a reference molecule which is a topoisomerase inhibitor, has less influence on Mdm2 and Pirh2 expressions in K562 cells. Strikingly, the PAC treatment has caused a dramatic fall in the expression levels of both Mdm2 and Pirh2 oncoproteins as compared to the untreated K562 cells (see Figure 7). These data partly suggest that PAC inhibits Mdm2 and Pirh2 oncoproteins which resulted in the rise in p53 tumour suppressor protein in K562 cells. On the other hand, the induction of p53 levels by CPT in K562 p53 null cells may be due to distinct mechanisms. Results of the overall *in-vitro* analysis suggest that PAC exhibited good anticancer activity against all the tested cancer cell lines. Enzyme binding studies are required to further validate the Mdm2 and Pirh2 promiscuous binding nature of PAC and its analogs. (Figures 11–13) (Astalakshmi et al., 2022).

**TABLE 4:** Description of the different cancer cell lines used to test anti-proliferation activity.

Cell line	ATCC	Cancer type	TP53 status	p53 variant	TP53_Allele type	Comments
**K562**	CCL-243™	Myeloid erythrocytic leukemia cell line	c.406 407ins1	p. Q136fs* 13 (sourced from COSMIC database)	Homozyg ous	p53-null Consists of mdm2 SNP309 T/G allele No overexpression of Mdm2

### 3.5 Molecular docking

Through our molecular docking experiment, we discovered that PAC is efficient. As a result, PAC had the highest ratings for Glide (−8.312 kcal/mol) and binding affinity −49.601 kcal/mol). The docked complex analysis revealed that the residues HIE96, TYR100 bonded with hydrogen bonding. Lys 173 was interacting with the ligand at the hydration site The Glide ratings for PAA were −7.216 kcal/mol, and the binding affinity was recorded as −42.371 kcal/mol. Analysis of the docked complex indicated the occurrence of hydrogen bonding between PAA and the residues TYR100 exhibited superior docking scores and binding affinity compared to PAA. Consequently, additional investigations and studies were conducted specifically focusing on PAC. (Table 5) (Vettoretti et al., 2016)**.**

**TABLE 5:** 2D interaction diagram of the Ligands with their interactions with the protein 3LBK.

Ligands	Two-dimensional inter-molecular interaction	Docking score	Active site residues
PAC	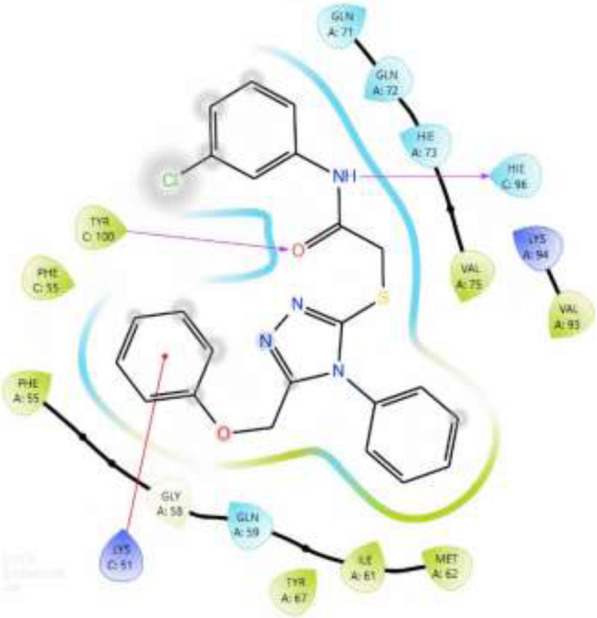 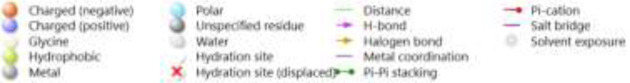	−8.574	HIE96, TYR100
PAA	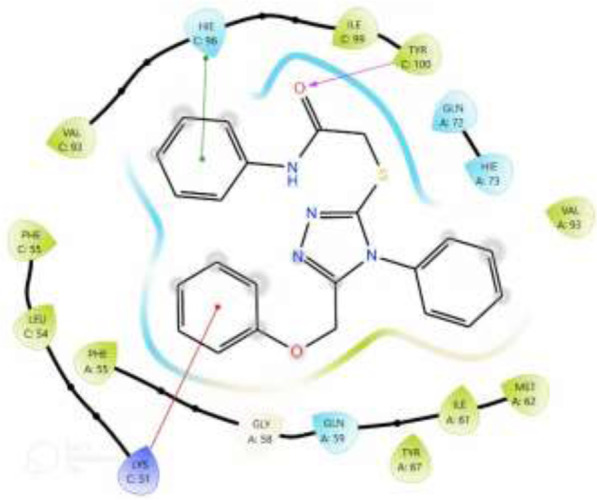 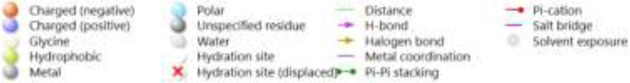	−7.096	TYR100

### 3.6 Molecular dynamic simulation

**FIGURE 14:**
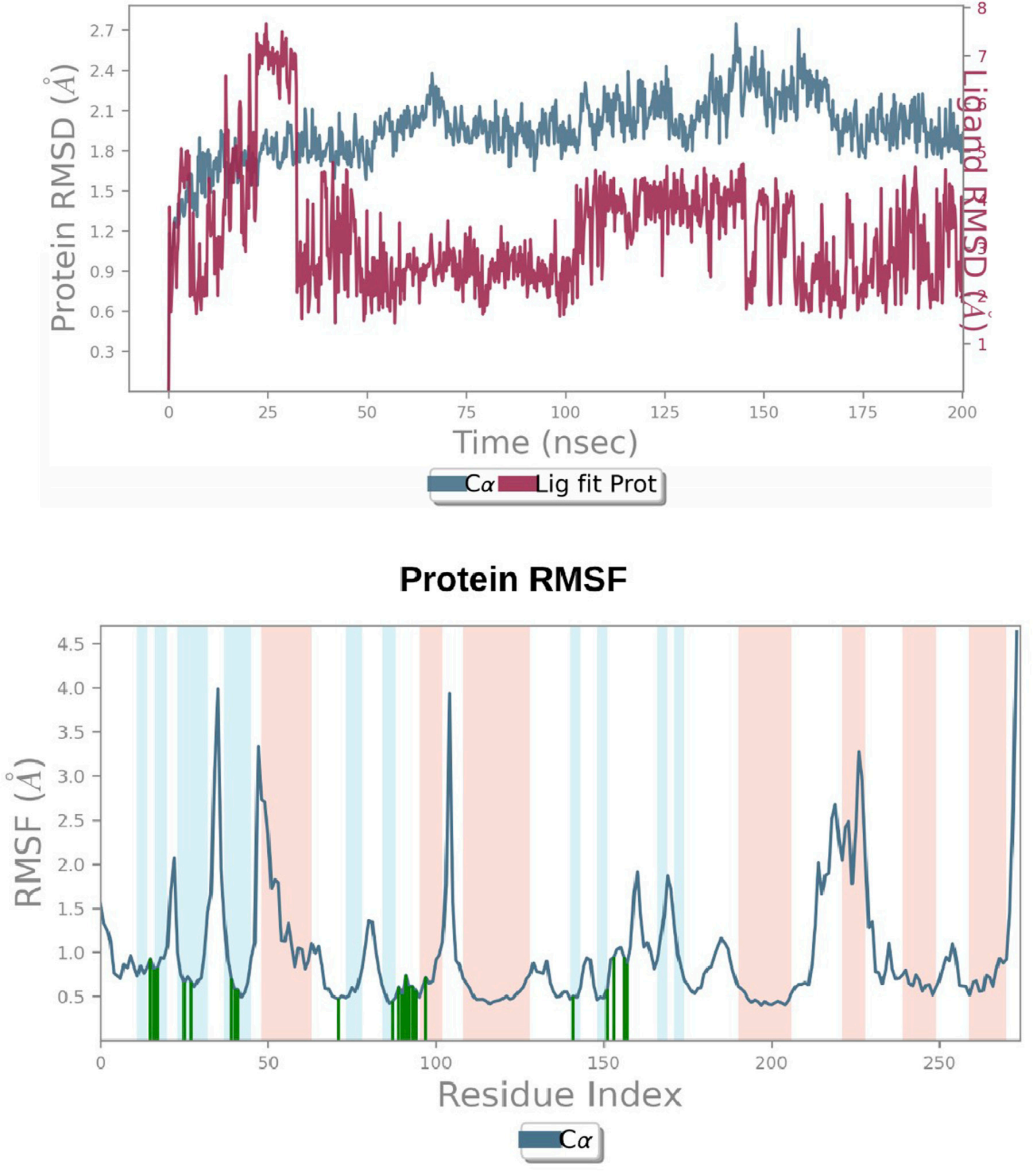
**(A)** RMSD graphs of Receptor-ligand complexes in MD simulations. **(B)** RMSF plot of Protein.

A simulation study was conducted using molecular dynamics (MD) to confirm the stability of the receptor-ligand complex, validate the predicted binding mode, and examine potential interactions. These aspects had been previously investigated through Glide XP docking. In the present investigation, we ran a 200 ns MD simulation of the PAC—3LBK complex. This extended simulation duration enabled the examination of molecular and atomic-level changes crucial for understanding the stability of the protein-ligand complex and the dynamic behavior of the ligand. Using the Desmond tool from the Schrödinger suite, a 200 ns simulation was conducted, and resulting trajectories were analyzed with the simulation interaction diagram panel to comprehend deviation fluctuation and intermolecular interactions. The root mean square deviation (RMSD) value was calculated to assess the deviation in the protein’s backbone throughout the 200 ns simulation period. The RMSD plot of the PAC—3LBK complexes are shown in Figure 12A, demonstrating slight conformational fluctuations in the complexes between 135 and 160 ns. RMSD levels range from 1.8 Å to 2.7 Å Around 8 Å. Similarly, there was a modest fluctuation in the ligand RMSD between 0 ns and 30 ns, with RMSD ranging from 0.6A to 2.7 around 2.1 Å. Despite these variations, the PAC—3LBK complex remained stable. The Root Mean Square Fluctuations (RMSF) aid in characterizing local protein alterations. These variations were used to identify the residue in the complex that contributes to structural fluctuations. The region of proteins was represented by peaks that vary throughout the process of simulation. The N and C terminal of protein tails changes more when compared to any other regions of protein. The alpha helices and beat chain are stronger than the unstructured regions of the protein and hence they alter less when compared to loop regions. The alpha and beat regions are represented in red and blue color. These regions were specified by helices or strands that continue 70% of simulation time. The proteins that are interacted with ligand are represented in green color with slight fluctuations, conforms the protein ligand complex are stable. (Figures 14A, B) (Li and Zhang, 2022).

## 4 Conclusion

In conclusion, our research focuses on developing and testing new anticancer drugs based on a 1,2,4-triazole scaffold. These chemicals were painstakingly crafted to alter the ubiquitination of the tumour suppressor protein p53, which is regulated by the E3 ubiquitin ligases Mdm2 and Pirh2. Notably, the principal chemical, PAC, demonstrated significant anti-proliferative effects across a range of cancer cell lines, with a particularly strong effect on leukemia cells. PAC modulates p53-mediated pathways by inhibiting Mdm2 and Pirh2, leading to the stabilization of p53.PAC promotes cell cycle arrest at the SubG0/G1, S, and G2 phases, indicating its ability to disturb normal cell cycle progression, according to cell cycle analyses. According to flow cytometry studies, PAC increases p53 expression while decreasing Mdm2 and Pirh2 levels in K562 cancer cells. A molecular dynamics simulation of PAC with the TP53 protein revealed sustained connections lasting 200 ns, highlighting its potential as a powerful anticancer therapy. Our complete technique combines manual design, organic synthesis, *in vitro* testing, and molecular dynamics modelling. This multidisciplinary strategy attempts to identify and characterize novel chemicals for cancer therapy. The compounds were created without the use of artificial intelligence, and the study highlights PAC’s potential as a game-changing anticancer treatment due to its multifaceted impact on cell cycle regulation and molecular interactions. This work contributes to the larger goal of producing effective and tailored cancer medicines, highlighting the need of varied approaches in drug discovery and development.

## Data Availability

The original contributions presented in the study are included in the article/Supplementary Material, further inquiries can be directed to the corresponding authors.
